# Investigation of the kynurenine pathway in *Indoleamine 2, 3 dioxygenase* deficient mice with inflammatory arthritis

**DOI:** 10.1007/s11248-013-9696-5

**Published:** 2013-02-17

**Authors:** Lukasz Kolodziej

**Affiliations:** 1 The Kennedy Institute of Rheumatology Division, Imperial College London, Arthritis Research UK Building, Charing Cross Hospital Campus, 65 Aspenlea Road, London, W6 8LH UK; 2 Department of Clinical Immunology Polish-American, Institute of Pediatrics, Jagiellonian University Medical College, Wielicka Steet 265, 30-663 Kraków, Poland

**Keywords:** IDO1, IDO2, Transgenic mice, Immunity

## Abstract

Tryptophan is an essential amino acid involved in the protein synthesis, cognition, and immunity. Oxidative catabolism of tryptophan is executed by the sets of biochemical reactions collectively referred to as the kynurenine pathway. In the immune system, two distinct enzymes, Indoleamne 2,3 dioxygenase 1 (IDO1) and Indoleamine 2, 3 dioxygenase 2 (IDO2) can initiate metabolic flux through the kynurenine pathway. Rheumatoid arthritis is an autoimmune disease driven by the exacerbated immune response towards self antigens and characterized by the chronic inflammatory reaction of the diarthrodial joints. Collagen induced arthritis (CIA) is an animal model of rheumatoid arthritis. Using CIA in wild type (WT) and mice deficient with Indoleamine 2,3 dioxygenase (Ido1KO), it was of interest to test the impact of Ido1 deletion on the concentration of tryptophan and its catabolites as well as on mRNA expression for other genes on the kynurenine pathway. Here, when compared with samples taken from naïve WT animals and those with CIA, it was found that only in the inguinal lymph nodes (iLN) taken from Ido1KO mice with CIA tryptophan concentration was significantly increased. In contrast, mRNA expression for *Ido2* was decreased in naïve as well as in the diseased iLN taken from Ido1KO mice. Deletion of *Ido1* and reduced mRNA expression for *Ido2* neither affected the concentration of the downstream metabolites of tryptophan nor mRNA expression for downstream genes on the kynurenine pathway in iLN. Moreover, the concentration of kynurenine in sera of mice with CIA was significantly decreased in Ido1KO mice with arthritis.

**Electronic supplementary material:**

The online version of this article (doi:10.1007/s11248-013-9696-5) contains supplementary material, which is available to authorized users.

## Introduction

**Fig. 1:**
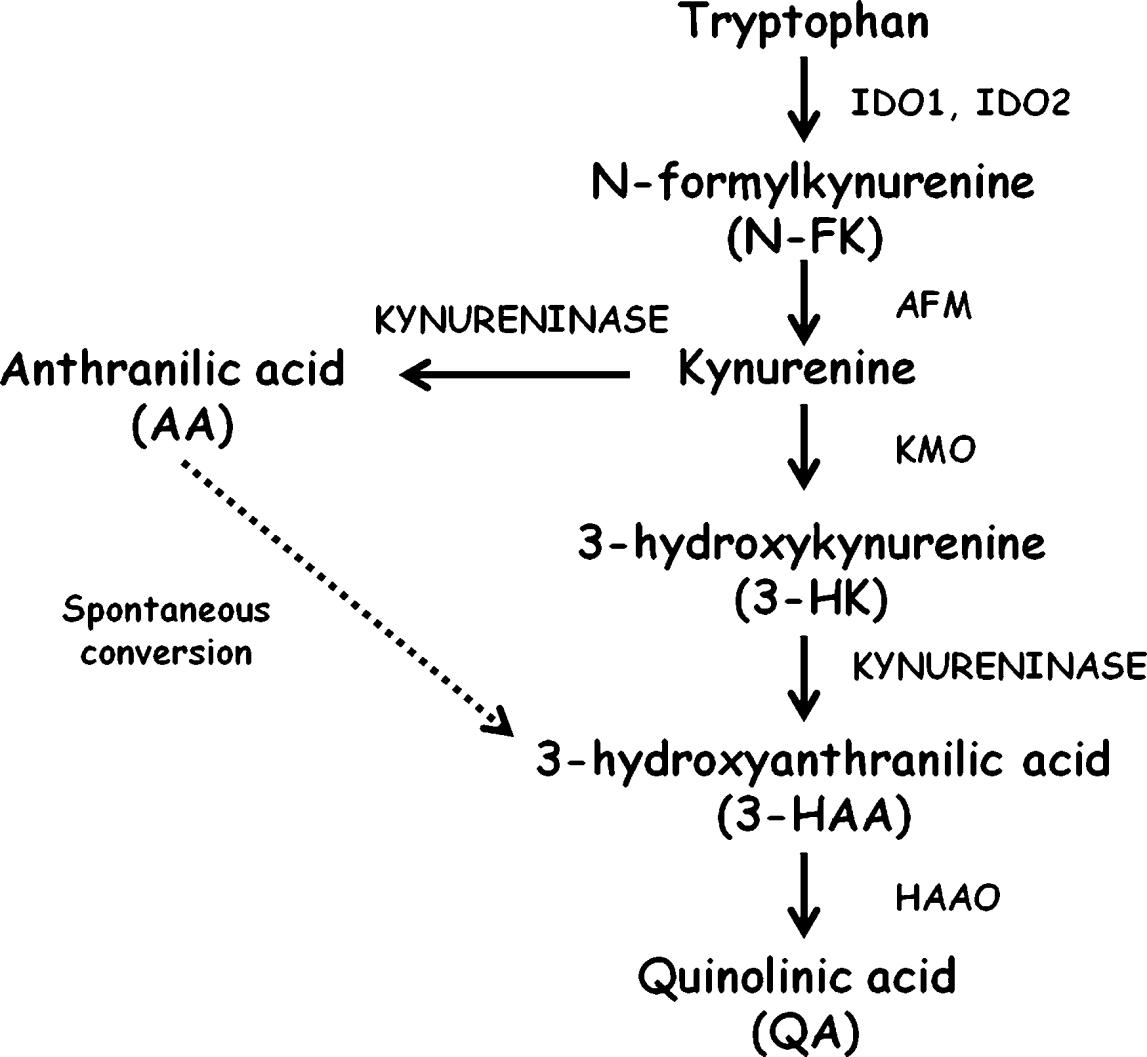
Simplified schematic representation of the kynurenine pathway. Enzymes involved in tryptophan metabolism via the kynurenine pathway: indoleamine 2,3 dioxygenase 1 (EC 1.13.11.17) *IDO1*, indoleamine 2,3 dioxygenase 2 (EC 1.13.11.52) *IDO2*, arylformamidase (EC 3.5.1.9) *AFM*, kynurenine 3-monooxygenase (EC 2.6.1.7) *KMO*, Kynureninase (EC 3.7.1.3), 3-hydroxyanthranilate 3,4 dioxygenase (EC 1.13.11.6) *HAAO*. Spontaneous conversion of AA into 3-HAA has been previously reported (Ueda et al. 1978)

Tryptophan is an essential amino acid implicated in the regulation of various biological processes including mood (Russo et al. 2003) and immunity (Moffett and Namboodiri 2003). Indoleamnine 2, 3 dioxygenase 1 (IDO1) is an initial enzyme which catalyses oxidative degradation of tryptophan via the kynurenine pathway (Fig. 1) (Hayaishi 1976). Hence, not surprisingly, IDO1 and the kynurenine pathway have been extensively investigated (Kolodziej et al. 2011). Therefore, mice deficient in *Ido1* (Ido1KO) are a vital tool in the research on IDO1 mediated tryptophan degradation (Baban et al. 2004). Moreover, another enzyme, Indoleamine 2, 3 dioxygenase 2 (IDO2), which is also able to catalyze oxidative catabolism of tryptophan via the kynurenine pathway, has been recently discovered (Metz et al. 2007). Interestingly, *Ido1* and *Ido2* are located on the same chromosome in the close proximity to each other (Ball et al. 2007). Hence, it could be predicted deletion of *Ido1* could be compensated by the increased expression of *Ido2* mRNA, resulting in the decreased concentration of tryptophan in Ido1KO mice despite inactivation of IDO1.

In the immune system, tryptophan has been implicated in the immune regulation (Moffett and Namboodiri 2003). The decreased concentration of this amino acids leads to cell cycle arrest (Frumento et al. 2002; Munn et al. 1999) and apoptosis (Fallarino et al. 2002). In addition, toxic byproducts of tryptophan degradation via the kynrenine pathway are potent inducers of apoptosis in T cells (Kolodziej et al. 2011; Terness et al. 2002). IDO1 mediated tryptophan catabolism has been also proven to be important in the biology of Th17 and Treg cells (Baban et al. 2009; Sharma et al. 2009). Th17 cells exacerbate inflammation (Weaver et al. 2006), whereas Treg cells exhibit potent anti-inflammatory activity (Zaiss et al. 2007). Interestingly, tryptophan starvation accompanied with accumulation of its toxic byproducts e.g. anthranilic acid (AA) and 3-hydroxyanthranilic acid (3-HAA) has been shown to abrogate function of Th17 cells in a dose dependent manner (Desvignes and Ernst 2009). In addition, the same conditions have been proven to promote the development of Treg’s (Fallarino et al. 2006). Thus, in the context of metabolic regulation, Ido1KO mice may be also a useful tool in the investigations focused on the reciprocal functional relations between Th17 and Treg cells.

Rheumatoid arthritis (RA) is an autoimmune systemic disease affecting around 1 % of the western population (Smolen and Aletaha 2009). In RA, activity of Th17 cells takes over an anti-inflammatory role of Treg cells and chronic inflammation of the joints progress (Sato et al. 2006). Collagen induced arthritis (CIA) is an animal model of RA (Inglis et al. 2007) driven by the execrated function of Th17 cells and abnormally low activity of Treg (Park et al. 2011). In Ido1KO mice with CIA the incidence of the disease has been found to be higher than in the wild type (WT) diseased mice (Criado et al. 2009). In addition, the severity of symptoms was higher in Ido1KO mice with CIA than in the WT animals with CIA (Criado et al. 2009). Thus, taken together, it was of interest to test if deletion of *Ido1* could be: (1) compensated by the increased mRNA expression for *Ido2* in iLN, (2) impact mRNA expression for downstream genes on kynurenine pathway (*Afm*, *Kmo*, *Kynu*, and *Haao*) in iLN during CIA, (3) influence the concentration of tryptophan and its anti inflammatory catabolites: kynurenine, AA, and 3-HAA in iLN from Ido1KO mice with CIA, and (4) coincide with reduced concentration of kynurenine in serum of Ido1KO mice with CIA.

## Materials and methods

### Animals, CIA development and tissue harvesting

All experimental procedures were approved by the UK Home Office. Adult C57BL/6J mice (aged 10–12 weeks) were used in experiments (Charles River, UK). CIA was induced as previously described (Inglis et al. 2007). Ido1KO mice were kept and bred in the Biological Service Unit in the Kennedy Institute of Rheumatology. Mice were humanely sacrificed and lymph nodes were immediately frozen and kept at −80 °C.

### HPLC analysis and kynurenine measurements

Concentration of tryptophan, AA, and 3-HAA was determined with HPLC method. The HPLC system (UltiMate 3000) was provided by Dionex, UK. All chromatographic procedures were performed in 37 °C, with C18 column (Acclaim 120, Dionex, UK) 3 μm, 120 Å; 4.6 × 150 mm, and injection volume of 10 μL. Tryptophan concentration was determined by HPLC with fluorescence detection (excitation λ = 284 nm; emission λ = 365 nm). The mobile phase (1 ml/min flow rate) consisted of 50 mM acetic acid, 100 mM zinc acetate, and 3 % acetonitrile). Concentartion of AA and 3-HAA was determined by HPLC with fluorescence detection (excitation λ = 320 nm; emission λ = 420 nm). The mobile phase (1 ml/min of flow rate) consisted of 25 mM sodium acetate (Sigma), 1 mM acetic acid (pH5.5). Kynurenine concentration was assessed by a colorimetric assay (Hara et al. 2008).

### RNA isolation, preparation of cDNA, and qRT-PCR

RNA was extracted using RNA-Stat60 reagent (AMS Biotechnology) according to the manufacturer’s instructions. cDNA was transcribed using the Applied Biosystems Reverse Transcription System. The total volume of qRT-PCR reaction was 10 μl. TaqMan primer probes were provided by Applied Biosystems UK. List of primers is shown in the supplementary table 1. PCR reaction was performed in a Corbett Rotor-gene 6000 thermocycler (Corbett Lifesciences, Sydney). mRNA expression was assessed by the ΔΔCt method.

### Statistical analysis

Data was statistically analyzed using Prism 4.03 software. *T* test was used to compare results between experimental groups.

## Results

**Fig. 2:**
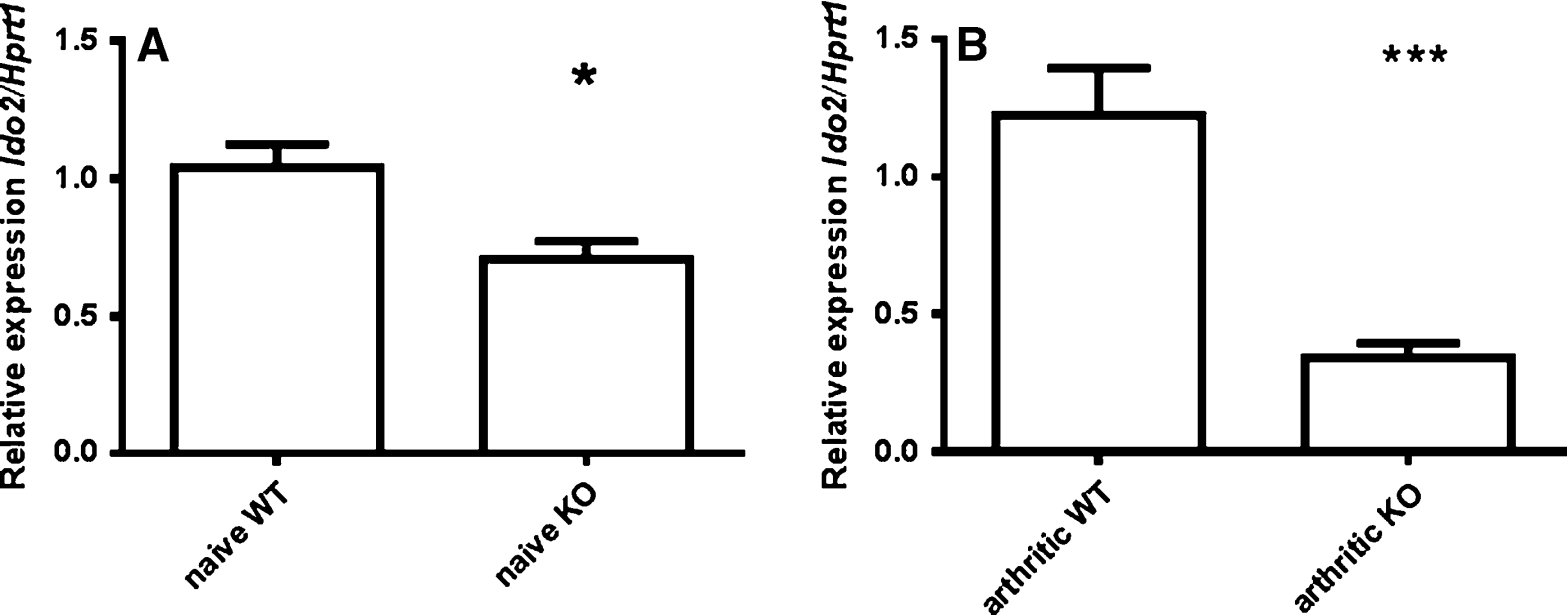
Decreased mRNA expression for *Ido2* iLN from naïve and arthritic Ido1KO mice in comparison with WT controls. CIA was induced in C57BL/6J mice (n = 5) and Ido1KO animals (n = 5). Ten days after onset of the disease have been spotted animals were sacrificed and iLN isolated. mRNA expression for *Ido2* was assessed using qRT-PCR and results were compared with naïve IDO1KO mice (n = 5) and naïve WT C57BL/6J animals (n = 5). Data was analyzed using *t* test. **a** Significantly decreased *Ido2* mRNA expression in iLN taken from naïve Ido1KO mice **b** significantly decreased *Ido2* mRNA expression in iLN taken from Ido1KO mice with CIA *p < 0.05; ***p < 0.001

In a first step, the concentration of tryptophan and kynurenine was measured in the iLN taken from naïve WT and healthy Ido1KO mice. However, it was found that neither the concentration of tryptophan nor the concentration of kynurenine was affected in iLN by the deletion of *Ido1*, Table 1. The lack of changes in the concentration of tryptophan and kynurenine in naïve iLN could be explained by the fact that loss of IDO1 function may be compensated by the increased expression of IDO2. Moreover, in contrast to what could be expected, it was found that mRNA expression for *Ido2* was significantly decreased (*p* < 0.05) in iLN taken from naïve Ido1KO mice (Fig. 2a).

**Table 1:** Concentration of tryptophan and kynurenine in iLN from WT and Ido1KO mice

Compound	Naive WT mice (nmol/g wet tissue)	Naive Ido1KO mice (nmol/g wet tissue)	Arthritic WT mice (nmol/g wet tissue)	Arthritic Ido1KO mice (nmol/g wet tissue)
Tryptophan	5.11 ± 0.85	6.07 ± 3.8	5.12 ± 0.4	8.68 ± 2.4*
Kynurnine	731 ± 250.7	618.7 ± 231	998 ± 709	589.7 ± 198.1

iLN were isolated from naive and mice with CIA of C57BL/6J strain and Ido1KO mice, n = 5 for each strain and conditions. The concentration of tryptophan was measured with HPLC method, whereas the concentration of kynurenine was measured with a colorimetric assay. Results were assessed using *t* test

* *p* < 0.05

In a next step it was of interest to test if CIA could impact the concentration of tryptophan and kynurenine in iLN taken from WT animals with CIA and diseased Ido1KO mice. Interestingly, the mean concentration of tryptophan was found to be significantly (*p* < 0.05) increased in iLN taken from Ido1KO mice with CIA in comparison with tissues harvested from WT arthritic animals, Table 1. Moreover, the concentration of kynurenine in iLN was not affected by CIA, Table 1. Next, mRNA expression for *Ido2* was assessed in iLN from arthritic mice. However, mRNA expression for *Ido2* was also significantly (*p* < 0.001) reduced in the iLN taken from Ido1KO mice with CIA in comparison with iLN taken from WT diseased mice (Fig. 2b).

It was also interesting to check if deletion of *Ido1* could impact the concentration of kynurenine in serum of arthritic mice. However, it was found that in Ido1KO mice with CIA the concentration of kynurenine was significantly (*p* < 0.001) decreased in comparison with WT mice with CIA. In sera of arthritic WT mice the mean concentration of kynurenine was 1.83 ± 0.24 μM. In contrast, in sera of Ido1KO mice with CIA the mean concentration of kynurenine dropped to 0.61 ± 0.11 μM.

In addition, it was also of interest to test if an inactivation of IDO1 and decreased mRNA expression for *Ido2* in iLN from Ido1KO mice with CIA could be compensated by the changes in the mRNA expression for downstream genes (*Afm*, *Kmo*, *Kynu,* and *Haao*) on the kynurenine pathway. However, no such effect was observed in iLN from Ido1KO mice with arthritis in comparison with WT mice with CIA (supplementary figure 1). Similarly, in iLN taken from neither naïve nor from the diseased Ido1KO animals, the concentration of kynurenine catabolites, AA and 3-HAA, was significantly changed (supplementary table 2).

## Discussion

The aim of this work was to investigate the impact of *Ido1* deletion on the concentration of tryptophan and its biologically active catabolites: kynurenine, AA, and 3-HAA in naïve iLN isolated from WT and Ido1KO mice as well as those taken from animals with CIA. In addition, the concentration of kynurenine was measured in sera of Ido1KO mice with CIA and WT arthritic animals. Metabolic data was also supported by the results showing mRNA expression for other genes on the kynurenine pathway in iLN upon *Ido1* deletion.

Interestingly, it was found that in naïve iLN taken from Ido1KO mice the concentration of tryptophan was not affected by the deletion of *Ido1*. In contrast, tryptophan was accumulated in iLN of Ido1KO mice upon immune challenge driven by CIA. Increased accumulation of tryptophan in iLN from Ido1KO mice during CIA may also emphasize a functional importance of IDO1 in the regulation of tryptophan concentration in the local tissue environment (Lob and Konigsrainer 2009; Lob et al. 2009). It is known that upon specific conditions (e.g. acidosis) tryptophan can be displaced from albumin (Cunningham et al. 1975). This process may impact the metabolic flux through the kynurenine pathway (Smith and Pogson 1980). Hence, increased concentration of tryptophan upon deletion of *Ido1* suggests that in deed IDO1 can regulate concentration of tryptophan in the local tissue environment. In addition, in the previous papers, I have shown that the full anti inflammatory potential of the kynurenine pathway is likely to be achieved upon coincidence between decreased concentration of tryptophan and accumulation of kynurenines in iLN (Kolodziej 2012) but not in the serum (Kolodziej 2013).

IDO2 is a relatively recently discovered enzyme which can also mediate oxidative catabolism of tryptophan (Metz et al. 2007). However, physiological importance of this enzyme remains elusive. Thus, Ido1KO mice could be a useful tool in the dissection of IDO2 role in the regulation of tryptophan catabolism. Moreover, here, it has been found that mRNA expression for *Ido2* was decreased in the iLN taken from naïve Ido1KO mice as well as those with CIA. Hence, Ido1KO mice may not be particularly suitable for investigations of a physiological role of IDO2.

Here, it has been also found that deletion of *Ido1* and significantly reduced *Ido2* mRNA expression did not result in the abnormally changed concentration of kynurenine and its anti inflammatory catabolites: AA and 3-HAA in the naïve iLN. Similarly, mRNA expression for downstream genes on the kynurenine pathway: *Afm*, *Kmo*, *Kynu*, and *Haao* were found to be not changed in iLN taken from Ido1KO mice with CIA. However, it has to be also acknowledged that the kynurenine pathway also consists of metabolic branches on which biologically active metabolites have been produced (Amori et al. 2009). It has been shown that kynurenic acid, derived from kynurenine, influenced leucocytes (Barth et al. 2009). Moreover, due to technical and financial limitations in this project, it was not possible to investigate metabolic flux through branches on the kynurenine pathway.

The concentration of kynurenine was discovered to be significantly decreased in sera of Ido1KO mice with CIA. This observation may be explained by two distinct but not entirely exclusive possibilities. It may be that upon immune challenge deletion of *Ido1* is sufficient enough to cause a decreased concentration of kynurenine in serum but not in the iLN. Alternatively, it has been shown kynurenine can be actively transported into the T cells via the CD98 transporter (del Amo et al. 2008). Hence, under reduced rate of kynurenine anabolism, this process could account for decreased concentration of kynurenine in serum (Kolodziej et al. 2011). Moreover, this theory needs to be experimentally tested yet.

Nonetheless, taken together, in this study it was shown deletion of *Ido1* alongside with decreased *Ido2* mRNA expression resulted in the accumulation of tryptophan in the iLN and reduced concentration of kynurenine in sera of transgenic animals with CIA. These results support the importance of IDO enzymes in the regulation of tryptophan concentration in the local tissue environment and immune system.

## Electronic supplementary material

Below is the link to the electronic supplementary material.

Supplementary material 1 (DOC 761 kb)
