# Supplementary material for: Investigation of the kynurenine pathway in Indoleamine 2, 3 dioxygenase deficient mice with inflammatory arthritis
Source: Transgenic Res. 2013 Feb 17;22(5):1049–54. doi: 10.1007/s11248-013-9696-5 (PMC3781305; doi:10.1007/s11248-013-9696-5)
Supplement: Supplementary file 1 — Supplementary material 1 (DOC 761 kb) [file 11248_2013_9696_MOESM1_ESM.doc]

**Supplemntary materials**

**Supplementary table 1**

List of genes and TaqMan probes with their reference numbers bought from Applied Biosystems, UK applied for qRT-PCR analysis

| Gene | Gene |
| --- | --- |
| *Hprt1* | Mm00446968_m1 |
| *Ido1* | Mm00492586_m1 |
| *Ido2* | Mm00524206_m1 |
| *Afm* | Mm00510774_m1 |
| *Kmo* | Mm00505511_m1 |
| *Kynu* | Mm00551012_m1 |
| *Haao* | Mm00517945_m1 |

**Supplementary table 2 Concentration of AA and 3-HAA in the iLN**

iLN were taken from naive or CIA mice of C57BL/6J strain (n=5) and Ido1KO mice (n=5). Concentration of AA and 3-HAA was measured with HPLC method. Results were assessed using t-test. However, no significant changes could be detected between samples.

| **Compound** | **Naïve WT** | **Arthritic WT** | **Naïve KO** | **Arthritic KO** |
| --- | --- | --- | --- | --- |
| **AA**  **nmol/g of wet tissue** | 1.37 ±0.85 | 0.76 ±0.25 | 0.61 ±0.26 | 0.68 ±0.18 |
| **3-HAA**  **pmol/g of wet tissue** | 121.38 ±40.63 | 155.7±40.83 | 75 ±39 | 102.13 ±15.61 |





**Supplementary figure 1 mRNA expression for the kynurenine pathway enzymes in iLN from Ido1 KO mice with CIA**

CIA was induced in Ido1KO mice and C57BL/6J. Ten days after onset of the disease were spotted animals were sacrificed and iLN taken. mRNA expression for **A)** Afm **B)** Kmo, **C)** Kynu, and **D)** Haao was assessed with qRT-PCR. Results were assessed using t-test. However, no significant differences were found.
